# Clinician perspectives on inpatient cystatin C utilization: A qualitative case study at Mayo Clinic

**DOI:** 10.1371/journal.pone.0243618

**Published:** 2020-12-11

**Authors:** James Roland Markos, Karen S. Schaepe, Hilary R. Teaford, Andrew D. Rule, Kianoush B. Kashani, John C. Lieske, Erin F. Barreto

**Affiliations:** 1 Mayo Clinic Alix School of Medicine, Mayo Clinic, Rochester, MN, United States of America; 2 Robert D. and Patricia E. Kern Center for the Science of Health Care Delivery, Mayo Clinic, Rochester, MN, United States of America; 3 Department of Pharmacy, Cedars-Sinai Medical Center, Los Angeles, CA, United States of America; 4 Division of Nephrology and Hypertension, Mayo Clinic, Rochester, MN, United States of America; 5 Division of Epidemiology, Mayo Clinic, Rochester, MN, United States of America; 6 Division of Pulmonary and Critical Care Medicine, Mayo Clinic, Rochester, MN, United States of America; 7 Department of Laboratory Medicine and Pathology, Mayo Clinic, Rochester, MN, United States of America; 8 Department of Pharmacy, Mayo Clinic, Rochester, MN, United States of America; University of Liège, BELGIUM

## Abstract

**Introduction:**

Serum creatinine (SCr) testing has been the mainstay of kidney function assessment for decades despite known limitations. Cystatin C (CysC) is an alternative biomarker that is generally less affected than SCr by pertinent non-renal factors in hospitalized patients, such as muscle mass. Despite its potential advantages, the adoption of CysC for inpatient care is not widespread. At one hospital with CysC testing, we demonstrated a significant rise in non-protocolized use over the last decade. This study uses qualitative methods to provide the first report of how clinicians understand, approach, and apply CysC testing in inpatient care.

**Methods:**

Fifteen clinicians from various disciplines were interviewed about their experience with inpatient CysC testing. The semi-structured interviews were audio-recorded, transcribed verbatim, and analyzed thematically using a phenomenological approach.

**Results:**

Knowledge and confidence with CysC varied greatly. Clinicians reported first learning about the test from colleagues on consulting services or multidisciplinary teams. The majority believed CysC to provide a more accurate measure of kidney function than SCr. Common scenarios for CysC ordering included medication dosing, evaluation of acute kidney injury, and a thorough evaluation of kidney function in patients with risk factors for an altered SCr. Facilitators for ordering CysC included the availability of rapid results turnaround and the automated calculation of glomerular filtration rate based on the biomarker. Barriers to use included a lack of education about CysC, and the absence of an institutional protocol for use.

**Discussion:**

Clinicians at our site decided independent of institutional guidance whether and when CysC added value to patient care. While the majority of study participants indicated advantages to rapid turnaround CysC testing, its use depended not just on the features of the specific case but on clinician familiarity and personal preference. Findings from this research can guide the implementation and expansion of CysC testing.

## Introduction

Assessment of kidney function and glomerular filtration rate (GFR) using endogenous kidney biomarkers plays a critical role in healthcare decision-making. In the hospital, these biomarkers can identify acute kidney injury, enhance the safety of medication dosing, assist with the decision to use radiocontrast media, and optimize fluid and hemodynamic status [1–5]. The primary biomarker used to estimate GFR (eGFR) is the serum creatinine (SCr). SCr has many strengths, including its widespread availability worldwide, low cost, clinician familiarity with the test, and its inclusion in nearly all processes of care, including clinical decision support, automated eGFR calculations, and drug information compendia. However, SCr exhibits a predictable lag from the onset of kidney damage and is affected by many nonrenal factors. As a terminal byproduct of skeletal muscle catabolism, age, sex, weight, height, and muscle mass each influence SCr concentrations, independent of underlying GFR [6–9]. Among hospitalized patients, non-renal determinants of SCr are especially frequent including altered dietary protein intake, malnutrition, and skeletal muscle wasting. For these reasons, adjuncts or alternatives to SCr have been sought.

One of the most promising new biomarkers of kidney function is serum cystatin C (CysC). CysC is a low molecular weight protein released from all nucleated cells that is freely filtered at the glomerulus and not systemically reabsorbed or actively secreted in the tubules [10, 11]. Unlike SCr, CysC is less affected by variation in muscle mass, sex, or weight [12, 13]. Still, CysC concentration can be affected by non-renal factors, including inflammation, altered cell turnover, and corticosteroid use [14, 15]. In stable ambulatory patients, eGFR with CysC in combination with SCr better predicted measured GFR than did eGFR with either biomarker alone [16]. In hospitalized patients, eGFR with CysC is at least as accurate as eGFR with SCr, and in certain circumstances, such as in the setting of acute kidney injury, amputation, quadriplegia, and in the critically ill, it may be more accurate [3, 17–23].

Despite the potential advantages of using CysC in the hospital as an adjunct to SCr, it is not yet widely available with a reasonable turnaround time. We recently demonstrated in a statewide survey that while CysC was available for use at 80% of acute care hospitals, same-day turnaround of results was present in only 3% [24]. Unlike the outpatient setting where CysC might be used as a confirmatory test for diagnosis of chronic kidney disease, in the inpatient setting where patient status is dynamic, delayed test turnaround effectively precludes its routine use. While access to CysC testing may be sparse, when available, it appears there is a trend toward increasing use. In a recent survey of clinicians managing AKI, 49% of respondents indicated novel biomarkers could be combined with SCr to inform care. CysC was reported as the most commonly used novel biomarker among clinicians [25]. At our institution, the Mayo Clinic, where rapid turnaround testing and automated eGFR reporting are available, a 19-fold increase in non-protocolized CysC utilization was observed over the last 8 years [24]. The explanation for this rise, practitioner familiarity and confidence with CysC, and approach to utilization and interpretation remain unstudied. The purpose of the present study was to enrich our previous quantitative findings with qualitative data from frontline clinicians to answer the research question, “How do clinicians understand and approach the use of CysC testing in the hospital setting?”

## Methods

This qualitative study is the second part of an explanatory sequential mixed-methods evaluation to describe the use of CysC in the hospital setting. The first part (quantitative) has been previously published [24].

### Site selection and study context

We selected Mayo Clinic in Rochester, Minnesota for our case study. Our goal in selecting one site was to establish an initial baseline for how the test is integrated into one hospital where rapid results are available. Mayo Clinic is a large academic medical center with 176 adult intensive care unit beds. It is one of the few hospitals in Minnesota that has rapid-turnaround CysC testing available (≤3 hours from sample collection) and the test is integrated into routine use. Rapid turnaround CysC testing has been available at this site for over 18 years but is not part of a formal clinical protocol. It is ordered at the discretion of the care providers. The tests are performed 24-hours per day, 7-days per week, and the results are reported in the electronic health record with the CysC concentration and automatic eGFR derived from the CKD-EPI eGFR_cystatin C_ equation (mL/min/1.73m^2^) [16]. Our previous quantitative research found that over a nine year period the CysC test was ordered by providers across specialties, with only 42% (3,032/7,162) of tests involving a nephrology consult [24]. The present study was approved by the Mayo Clinic Institutional Review Board (IRB) (protocol #19–008158) and reported in accordance with the consolidated criteria for reporting qualitative research (COREQ) [26].

### Theoretical framing

We used descriptive phenomenology (DP) as the methodological framework for understanding clinicians’ thoughts about ordering and utilizing CysC. This is a step-wise approach especially useful for developing themes and simultaneously revealing underlying structures within the participant behavior data [27, 28].

### Sampling

Our sampling strategy was purposive and required study participants to have both access and occasion to order CysC testing in the hospital setting. Clinicians were recruited from different specialty areas and different levels of experience and training (Table 1). Non-nephrology providers were purposefully oversampled to reflect the breadth of clinicians that interface with kidney assessment in hospital practice. In our previously published quantitative evaluation, nephrology specialty consultation co-occurred with a minority of CysC tests. Senior nephrologists were study co-investigators. The study PI was a pharmacist at Mayo Clinic with expertise in kidney assessment including with the use of CysC in the hospital [29–31].

**Table 1:** Clinician characteristics and perspectives on cystatin C testing.

ID	Title	Gender	Years in Practice	Clinical scenarios where CysC is used	Do CysC results alter/inform care plan?	Role in decision-making	Perceived need for/utility of CysC testing
	Internal Medicine						
ID1	Physician (attending)	Male	33	Polypharmacy with concern for toxicity, malnutrition, low muscle mass	Yes, rarely	Confirmatory	Useful in niches
ID2	Physician (attending)	Male	21	Low muscle mass, amputees, paraplegia, elderly, acute kidney injury	No	Confirmatory	Ambivalent
ID3	Physician (resident)	Male	0 (PGY-3)	Low muscle mass, elderly	Yes, rarely	Confirmatory	Useful
ID4	Physician (resident)	Male	0 (PGY-1)	Concern about acute kidney injury, low muscle mass, drug dosing	Yes, rarely	Refine drug dosing	Useful in niches
ID5	Physician (resident)	Male	0 (PGY-1)	Poor muscle mass, at the direction of other team members	No	Confirmatory	Uncertain
	ICU/Critical Care						
ID6	Physician (attending)	Male	18	Low muscle mass, elderly, low urine output with preserved serum creatinine	Yes	Confirmatory	Useful
ID7	Physician (attending)	Male	8	Low muscle mass, elderly, general concern about kidney function, drug dosing	Yes, rarely	Confirmatory	Useful in niches
ID8	Nurse Practitioner	Female	1.5	Elderly, treatment with antibiotics to assess drug dosing	Yes	Confirmatory	Useful
ID9	Physician (attending)	Female	5	At the direction of other team members, for drug dosing	No	Confirmatory	Uncertain
	Pharmacy						
ID10	Pharmacist	Male	13	Long hospitalization, low urine output, elderly, drug dosing	No	Confirmatory	Not necessary
ID11	Pharmacist	Male	5.5	Malnutrition, low muscle mass, general concern about kidney function, chronic kidney disease	Yes, rarely	Confirmatory	Useful in niches
ID12	Pharmacist	Male	16	Low muscle mass, drug dosing, for trending kidney trajectory	Yes	Confirmatory	Yes
	Infectious Disease						
ID13	Physician (attending)	Male	9	Frailty, low muscle mass, drug dosing	Yes, rarely	Confirmatory	Useful in niches
ID14	Physician (attending)	Male	8	Drug dosing	Yes, rarely	Refine drug dosing	Useful in niches
	Nephrology						
ID15	Physician (attending)	Male	10	High and low muscle mass, elderly, drug dosing, intermediate tool to alleviate the need for measured GFR	Yes	Complementary	Useful

### Data collection

Interviews were conducted from October 2019-February 2020 using the semi-structured interview guide in S1 Appendix. We estimated 20 interviews may be needed to achieve thematic saturation; however, after 15 interviews no new themes relevant to our research question and central domains emerged. Thus, the team met and decided to conclude further recruitment. Twelve of the interviews were conducted in-person in private meeting rooms at the hospital and three were conducted by phone. Prior to each interview, participants were recorded expressing verbal agreement to an IRB-approved consent form. The average interview length was 31 minutes, and all were transcribed verbatim. The first five interviews were conducted jointly by the study PI (Barreto) and a sociologist co-investigator (Schaepe) to ensure the guide adequately captured key technical features of the CysC testing process as well as the thought process related to decision-making. A review of the first five interviews led to modest guide refinements and Dr. Barreto and Dr. Schaepe conducted the remaining ten interviews individually. Interviews with participants who had a previous working relationship with Dr. Barreto were conducted by Dr. Schaepe.

### Data processing and analysis

The data was held confidential (shared only among the team members) and partially de-identified for analysis. Knowledge of the participants’ training, specialty, and years of experience was retained to provide necessary contextual information. Themes were identified both in advance of data collection (*a priori*) and also from review of the empirical data (*emergently*). Three *a priori* domains of interest that framed the research were: (1) clinician knowledge and confidence with CysC testing; (2) clinician approaches to CysC test use and interpretation in hospital practice; and (3) objective assessment of the institutional barriers and facilitators of CysC utilization. Throughout the data collection process, Dr. Barreto and Dr. Schaepe met regularly and shared impressions, discussed *emergent* themes in the interviews, and developed a codebook to discern the relationship between emergent themes and the three original domains of interest. When data collection concluded and all of the interviews had been transcribed, a third team member (Markos), read the transcripts independently and developed a descriptive summary of each domain of interest using Gale’s framework methodology [32]. The framework was used to generate a comprehensive table of responses by participant. The structured discussion of themes and subsequent axial coding were used to construct linkages between different themes in the data. The final phase of analysis involved returning to the original transcripts and audiotapes to ensure the identified themes and the selected quotes relayed the data accurately and reflected the original context in which comments were made [28]. All members of the investigative team evaluated the interpretations for completeness.

## Results

Overall, 17 clinicians were invited via email by the study PI (Barreto) to participate. Of these, 15 clinicians agreed to participate, and 2 declined due to time constraints. Our sample included attending physicians, resident physicians, pharmacists, and one nurse practitioner drawn from five core groups: internal medicine, critical care, pharmacy, infectious diseases, and nephrology (Table 1). Participant time in clinical practice ranged from 1 to 33 years. Six individuals were explicitly recruited because of work experience at another hospital to provide comment on institutional differences in kidney assessment across hospitals.

### Domain 1: Knowledge and confidence with cystatin C testing

Clinicians were very comfortable with SCr, the traditional test for kidney function. They described the test’s limitations effortlessly and articulated the ways they worked with or around readings they suspected to inaccurately reflect a patient’s true kidney function (e.g., in patients with low muscle mass). Only occasionally did a SCr result prompt ordering of a CysC test. Individual respondents noted ordering CysC anywhere from multiple times per week to several times per month to less than ten times during their career or in one case, never.

There was substantial variation among clinicians in terms of their knowledge and overall confidence with the CysC test, which we roughly classified along a continuum of “novice” to “expert” (Table 1). *“Experts”* had extensive experience ordering CysC, demonstrated an ability to interpret and apply it to patient care, reconciled disparate CysC and SCr results, and were able to identify key nonrenal determinants of CysC. Three pharmacists and five physicians (53% of the sample), fell into in the expert category, which was associated with a greater degree of overall clinical experience. Despite relatively robust knowledge, most interviewees were unable to articulate the specific eGFR equations or the units of measure (mL/min or mL/min/1.73m^2^) for CysC or SCr-based estimates of kidney function used institutionally. Most individuals were also unable to describe when one equation or unit of measure would be preferred. The knowledge and confidence of the nephrologist interviewed far exceeded that of all of the other study participants.

On the other end of our continuum were *“novices*.*”* These individuals reported ordering CysC only when instructed by a supervisor or consulting service. They relied exclusively on the automated eGFR result to interpret CysC and could not identify any of the non-renal determinants of CysC. Two participants, a PGY-1 internal medicine resident and an early career critical care physician, fell into this category. Between these two poles (novice to expert) were five individuals we would characterize as *“proficient*.*”* That is, these individuals exhibited a moderate familiarity with considerations for use of CysC and were reasonably comfortable using CysC to aid care decisions. These individuals explained the purpose of CysC was to broadly navigate drug dosing or care management decisions with little interest in precise numerical information in the lab results.

Regardless of level of expertise, all participants articulated a belief that CysC was generally more accurate for hospitalized patients than SCr, irrespective of patient demographics or health state. CysC ordering was triggered by concerns about SCr accuracy and a need for additional information on kidney function to guide care. No participants described situations where they would ignore a CysC result if it seemed abnormal or spurious.

In addition, we found that none of the clinicians had received formal training for use of the CysC test. A few became aware of the test’s existence during medical training or working somewhere else, but all indicated their first experience ordering or being part of a team using the CysC test was at Mayo Clinic. No participant had rapid turnaround CysC accessible in their practice at an outside institution. One person noted learning about it from attending a critical care lecture:

*“I probably started ordering it more after that Grand Rounds because I understood it*.*”*

                         (*ID8*, *Nurse Practitioner*, *ICU*)

The factor most determinant of CysC use was previous peer observation or discussion with colleagues. Individuals observed others ordering CysC, talked about ordering CysC during rounding, and recalled consulting nephrologists and pharmacists who recommended the test, all of which raised awareness and led in some instances to deliberate self-education about CysC:

*“Pharmacists here will occasionally ask for it and that’s where I became familiar with it*, *looked it up*, *and started to use it*.”

                         (*ID7*, *Attending Physician ICU)*

### Domain 2: Approaches to cystatin C utilization

#### Clinical scenarios for use

Participants noted several circumstances for CysC use. The three most common scenarios were for: (1) clarifying GFR in light of SCr confounders, (2) assessing kidney insults and acute kidney injury (AKI), and (3) dosing of renally-eliminated medications.

#### Clarifying GFR in light of SCr confounders

Clinicians described the generic scenario of patients coming into the hospital with low muscle mass as a condition where the true GFR may be lower than predicted by SCr. CysC was mentioned as helpful for patients with low muscle mass due to reasons including old age, cachexia, malnutrition, and cerebral palsy. One physician described the decision-making process:

*“I had a patient whose SCr was 0*.*8*. *So based on the SCr and the patient’s age*, *the computer would say that their eGFR was >60*, *a good number*. *And I knew that this was a lie—a bald-faced lie—because the patient had been through a lot recently*, *a lot in the distant past*, *had had several rounds of chemotherapy*, *and that the best explanation for the low SCr—or the normal-appearing SCr—was reduced muscle production*. *So I thought it was very helpful for me to be able to send the CysC*, *which said that her eGFR was 20*, *doesn’t require renal replacement therapy*, *but 20 is low enough that you would start to consider a run of dialysis during the hospitalization*.*”*

                       *(ID7*, *Attending Physician*, *ICU)*

Clinicians often had an intuition that SCr results were inaccurate and ordered the test to add objective data to reinforce this belief. An internal medicine resident (ID2), recalled a patient with pneumonia and cerebral palsy in whom he ‘knew’ the SCr based on the patient’s weight, age and comorbidities was not going to be accurate so he ordered the CysC for confirmation. As another clinician noted, CysC provides quantitative data regarding the eGFR even in the presence of non-renal determinants of SCr:

*“What’s so nice about the CysC is I often have an instinct that the kidney filtering is abnormal*, *even when the SCr doesn’t show that*.*”*

                         *(ID8*, *Nurse Practitioner*, *ICU)*

#### Assessing kidney insults and acute kidney injury

For many in critical care, CysC is viewed as one piece of information in a larger context of clinical indicators, including the SCr, urine output, hemodynamics, and clinician gestalt. The individual most knowledgeable about CysC described how estimated GFR by CysC could clarify procedural decisions and alter approaches to clinical management:

*“…patients we were going to dialyze*, *but then we decided not to dialyze because we think that maybe there’s some recovery*.*”*

        s                   *(ID15*, *Nephrologist)*

These more nuanced management strategies based on CysC results (i.e., dialysis management, kidney biopsy evaluation) were unique to the nephrologist. This is to be expected given that nephrologists are primarily responsible for such choices in practice.

Another related aspect of the test described in this domain was use in anticipation of potential kidney insults throughout the patient’s hospitalization and as an early indication of changes in kidney recovery or deterioration:

*“In addition to ordering SCr*, *[I] order CysC on top of it for the purpose of trying to better understand the trajectory of an AKI to see whether or not acute worsening or progression could be going on*.*”*

                         *(ID3*, *PGY3*, *Internal Medicine)*

#### Renal dosing of medications

Medication dosing was mentioned as the most clear-cut reason for performing a CysC test. Dosing considerations were seen as particularly critical when caring for elderly patients with low muscle mass, and those with malnutrition, chronic kidney disease, or multiple complex conditions. Precise knowledge about kidney function was also mentioned as important when prescribing nephrotoxic drugs, such as vancomycin.

*“Before we make a big commitment to a dose or after we’ve given a dose and found a level that’s surprising*, *we want more information about the kidney function*. *That’s another place CysC can be very helpful*.*”*

                       *(ID7*, *Attending Physician*, *ICU)*

#### Interpretation of test results

As alluded to in the quotes above, ordering CysC was never done in a vacuum; the interpretation of results was always compared to SCr and in the context of other information about the patient. CysC was ordered to confirm or refute the perceived accuracy of SCr. When the findings were concordant (i.e., similar to SCr), it was described as the last data needed before proceeding with the patient care plan. When the measures were discordant, CysC was taken as a more reliable measure, but clinicians varied in how they reconciled the differences. When we asked, “If you obtained an eGFR of 30mL/min with one tool and 80mL/min with the other (a significant discrepancy in kidney function for drug dosing), how would you proceed?” The majority favored the CysC-based estimate, especially if it yielded the lower eGFR. However, one physician said:

*“I just use the lower number […] I think*, *basically*, *I just- being a conservative person*, *I just look at the worst-case scenario and go with that*.*”*

                 *(ID2*, *Attending Physician*, *Internal Medicine)*

When we asked if they ever ‘averaged the two readings’ (analogous to using a combined eGFR equation such as with the CKD-EPI equation) or selected a mid-point estimate from the two results, only one participant noted this was how they would tend to resolve the discrepancy.

Ultimately, a theme across interviews was that kidney evaluation was dependent on numerous factors and was never a matter of relying on one data point. Both the ordering and interpretation of the results especially in the intensive care unit are done in an effort to reduce uncertainty about true kidney function based on vague or discrepant information about the patient.

*“We don’t fixate on one item ever*. *It’s always a combination of different items*, *even if we are evaluating*. *So for kidney function*, *yes*, *we’ll look probably at the cystatin C and the GFR that’s calculated*, *the creatinine*, *but also urine output—so it’s never like*, *‘This is the most important thing for me to look at’*.*”*

                         *(ID9*, *Attending Physician ICU)*

A pharmacist (ID12) similarly remarked none of the labs are evaluated in isolation but instead, “you try to evaluate a pattern.”

### Domain 3: Barriers and facilitators to CysC utilization

The final domain focused on factors that clinicians felt made it easier or more difficult to order and use CysC (Table 2).

**Table 2:** Factors influencing cystatin C utilization.

Barriers	Facilitators
Lack of education about CysC	Rapid turnaround time (<3 h)
Fluency with serum creatinine vs. unfamiliarity with CysC	Automated eGFR reporting in EHR
Absence of institutional	Knowledgeable individuals/CysC advocates
practice guidance/policy	
Location of CysC results in EHR	Team-based, multidisciplinary care model
	Education about CysC (formal and informal)
	Ease of test/low patient burden (blood draw)

#### Facilitators

Facilitators for CysC testing included rapid test result turnaround, automated eGFR reporting in the electronic health record, easy access to team members knowledgeable about CysC, and the ease of adding the test to stored blood specimens without needing a new blood draw. Rapid turnaround of results was mentioned as essential for CysC to impact clinical decision making in the acute care setting. Without it, the findings from CysC would be as one intensivist noted, “clinically irrelevant.” Likewise, the automated eGFR reporting facilitated use because it translates CysC results into a scale, using numerical “language” clinicians are familiar with–the eGFR. As one interviewee explained:

*“I would have no idea how to interpret CysC without a GFR attached to it*.*”*

                         *(ID5*, *PGY1*, *Internal Medicine)*

Access to knowledgeable individuals comfortable with the test was a facilitator because it allowed an informal introduction to the test. Nephrologists and pharmacists were mentioned by several participants as a “go-to” person for CysC ordering and interpretation. One hospitalist went as far as to say the increase in CysC use at Mayo Clinic is driven by nephrology:

*“If you are following nephrology patients and they’re recommending it regularly because they’re really interested in it*, *I think you just sort of- it just gradually becomes sort of second nature*, *‘Oh I just better order this because somebody else did it before me and I don’t feel that I should go against that*.*’”*

                      *(ID1*, *Physician*, *Internal Medicine)*

The team-based multidisciplinary care model facilitated education, interpretation and review of CysC test usage and was an opportunity to understand how nephrologists and pharmacists thought about the test. As one physician explained:

*“In our multidisciplinary rounds*, *I think pharmacists are key because I do very much rely on their ability to help us with drug dosing*, *and if they think the CysC is helping them*, *I have no problem [ordering it]*.*”*

                       *(ID9*, *Attending Physician*, *ICU)*

#### Barriers

Barriers to CysC testing included a lack of formal education about CysC, the absence of institutional guidance or recommendations for using the test, and the actual location of CysC results in the electronic health record. Clinicians routinely compared SCr to CysC noting the relative gap in understanding of the numbers. Even two of the pharmacists (ID12, ID13) who fell into the CysC “expert” category noted being less comfortable with CysC in comparison to SCr. The lack of formal institutional guidelines regarding the test left each clinician to determine for themselves whether to use the test. As one intensivist noted:

*“For me*, *it [CysC] seems like something that I am interested in doing professionally*, *and I can imagine that maybe not everybody is*, *but it has been useful for me*.*”*

                       *(ID7*, *Attending Physician*, *ICU)*

Cost appeared to have little influence on CysC utilization. Some participants mentioned cost as a potential concern, but thereafter noted no impact on their ordering behavior presumably because in the scheme of ICU costs this test was very inexpensive.

*“I think there are times where you’re like*, *well- you’re always considering like*, *“Well I could order an ultrasound or I can order a CT*. *An ultrasound’s so much cheaper so you’re always- at some point*, *you’re questioning like*, *Is this the right test for the right cost for the patient” But I wouldn’t say that I’ve ever actually thought about that as relates to cystatin C*.*”*

                         *(ID8*, *Nurse Practitioner*, *ICU)*

### Emergent themes

#### Need for CysC testing

One curious theme we did not anticipate centered on provider views about the necessity of CysC testing for inpatient use. While all study participants recognized SCr had limitations, wished for a better test of kidney function, and believed CysC was a more reliable measure, views on the necessity of the CysC in the acute care setting remained mixed. Only one-third of participants (N = 5), in fact, explicitly indicated it as a valuable test and served an important role in some patient scenarios.

*“Most situations*, *maybe*, *you don’t need these tests*, *but I mean to have both the tests done*, *both*, *but I- so my view of- so in that situation where it’s really helpful*. *And there are other situations where it might not add much to the creatinine*, *but my own bias is that this test could be part of a panel*, *like you do- you’re doing a basic metabolic panel*.*”*

                         *(ID15*, *Nephrologist)*

Two interviewees thought it was rarely necessary, often redundant, and could even have a subtle corrosive impact on practice by normalizing over-testing and dulling clinical skills in discernment:

*“I feel they’re relinquishing their responsibility to critically think […] the great part about trying to figure out what a person’s actual kidney function is*, *is that you don’t need to have it to the nearest decimal point*.*”*

                         *(ID10*, *Pharmacist)*

The majority of respondents (N = 8), however, were ambivalent, leaning only slightly toward or slightly away from the value of the CysC test:

*“I look at it more as haphazard or maybe not necessarily a tiebreaker but additional information*, *and the information’s only useful if you act on it or if it can be helpful in your clinical decisions*. *[…] in theory*, *it’s the way to go*. *But I just*, *again*, *I haven’t really seen in practice that it always makes sense*.*”*

                   *(ID2*, *Attending Physician*, *Internal Medicine)*

The variability in views about the need for CysC may not in and of itself reflect the test’s objective value, but it does reveal how the test is perceived to add value. Perceptions are undoubtedly shaped by the fact that the test is used infrequently, primarily when there were doubts about the SCr reading and mostly to confirm or disconfirm the SCr reading. Very rarely did the findings change the planned direction for care of the patient.

#### Time-saving

One other theme bears mentioning, but for the most part never rose to the level of a “reason” when the clinicians discussed the circumstances for ordering the test: ordering the test helped expedite workflow in several ways. First the CysC test removed residual doubt about the actual kidney function. Several participants noted that findings from CysC lessened hesitation to proceed with a treatment plan by removing lingering concern about relying solely on a ‘gut feeling’. One clinician noted CysC could reconcile discrepant information from SCr more quickly and definitively and thus, minimize the influence of any personal biases:

*“[Otherwise] I might let my own personal feelings about wanting to be able to initiate something or not and wanting to be able to initiate something comes into play*.*”*

                      *(ID3*, *Resident*, *Internal Medicine)*

Second, one clinician recounted that he would often be in drawn-out discussions with team members about a patient’s kidney function and ordering the test cut through a lot of back and forth ‘opinion.’ He noted that:

*“I order it more often [than I used to] because I think it helps to clarify the conversation*. *It helps me to talk to my colleagues with an additional piece of data*.*”*

                       *(ID7*, *Attending Physician*, *ICU)*

Third, one other way clinicians reported ordering CysC was to expedite decisions because it reflected closer to real-time kidney function in comparison to SCr:

*“Cystatin C [helps] to kind of hopefully get a better*, *closer timeframe about what is their kidney function right now so we could help diagnose- or dose the antibiotics more appropriately*.*”*

                      *(ID4*, *Resident*, *Internal Medicine)*

While we would not argue based on these data that enhancing workflow is the primary reason for ordering the test, it does appear that it is an underappreciated motivation and likely woven in with clinical factors.

Quotations illustrating key findings for each study domain are summarized in Table 3.

**Table 3:** Examples and illustrative quotes for each domain of interest.

CysC testing domains	Illustrative quotes
**Domain 1: Knowledge and confidence**	
Knowledge and confidence among clinicians on a continuum from novice to expert.	***Novice*:** “I would never check a Cystatin C [without being directed by a senior attending]… maybe that’s because I lack education on it- when it is appropriate.” (ID5)
	***Expert***: “The level [of cystatin C] will increase if- when patient have cancer, active cancer, or they’re on high-dose steroids, for example, some thyroid dysfunction.” (ID15)
**Domain 2: Utilization and need**	
***2a*. *Clinical scenarios for use*.** Situations where clinicians felt cystatin C testing was helpful for hospitalized patients.	***Assessing kidney insults and monitoring AKI***: *“I’ve used it in this way- In addition to ordering creatinine*, *[I] order cystatin C on top of it for the purpose of trying to better understand the trajectory of an acute kidney injury to see whether or not acute worsening or progression could be going on*.*” (ID3)*
	***Renal dosing of medications***: “If [SCr and CysC} are different and if I’m dosing a medication… I’ll probably use the cystatin C in that case because I know it is a more accurate representation of where their kidney function is right now.” (ID2)
	“Before we make a big commitment to a [drug] dose or after we’ve give a dose and found a level that’s surprising, we want more information about the kidney function. That’s another place cystatin C can be very helpful.” (ID7)
	***Clarify GFR in setting of creatinine confounders***: *“Someone who is very frail and doesn’t’ have much muscle mass and maybe we’re overestimating their GFR by creatinine… maybe the Cystatin C gives us a better or another indicator of what their true renal function is*.*” (ID13)*
	*“Usually for patients that come in who are malnourished or don’t have much for muscle mass*, *I’ll recommend a cystatin C for them*.*” (ID11)*
	*“What’s so nice about the cystatin C is I often have an instinct that the kidney filtering is abnormal*, *even when the creatinine doesn’t show that*.*” (ID8)*
***2b*. *Perceived need for CysC testing*.** Degree to which clinicians felt CysC was necessary to evaluation of kidney function.	***The right tool for certain niche***: *“*Most situations, maybe, you don’t need these tests, but I mean to have both the tests done—my view is in that [you want it in that specific] situation where it’s really helpful.” (ID15)
	***Another data point*:** “I look at it more as haphazard […] not necessarily a tiebreaker, but additional information. And the information’s only useful if you act on it or if it can be helpful in your clinical decisions. "(ID2)
	***Best uses still need to be defined*:** “To me, the bigger [question] is… when, actually, is cystatin C useful in determining a clinical change in- or meaningful change in what I’m going to do.” (ID1)
**Domain 3: Barriers and facilitators to CysC utilization**	
Structural factors or personal competence facilitating or hindering test ordering.	***Facilitator****—Rapid turnaround*: Without rapid results the CysC test would be *“*clinically irrelevant.” (ID7)
	***Barrier****–Lack of knowledge*: *“*I would have no idea how to interpret cystatin C without a GFR attached to it, but that’s why I use it so rarely.” (ID5)

## Discussion

This study aimed to assess how clinicians understand and interpret findings from CysC testing in the hospital setting. Direct insights from clinicians yielded a more fine-grained picture of how the test is thought about, ordered, and used to inform treatment decisions. We also identified institutional barriers and facilitators to CysC utilization in the acute care setting. To our knowledge, no prior studies have attempted to understand CysC ordering in acute care using a qualitative research design. While our study was not intended to render a verdict on the appropriateness or need for CysC in hospitals, it does provide a baseline for characterization of its use and role at our institution.

What we learned is that while the test is ordered infrequently overall, it is used routinely by some clinicians and frequently by a few others. This is likely due, in part, to minimal formal education or training regarding CysC, variable knowledge and experience and, and general preference. Regardless of level of expertise, clinicians said they trusted the findings from the CysC test over SCr. CysC was ordered primarily when the observed SCr was suspected to reflect an over-estimation of true kidney function. Cases where this was common included the elderly, those with low muscle mass, malnutrition, CKD, and/or with complex conditions. It might also be ordered when the patient had been in the hospital for an extended period of time. Also the test was often ordered for drug dosing, particularly when there was a need for greater precision due to a narrow therapeutic index. Overall, the findings from the test were comparative: used to confirm or disconfirm the accuracy of the serum SCr.

Nephrologists and pharmacists exhibited the most comprehensive knowledge of CysC testing. Other clinicians had a more limited working knowledge. Some clinicians stated that they could not interpret CysC results without the automated eGFR calculation displayed in the electronic health record, indicating the need to strategically design electronic systems to remove barriers to CysC interpretation for “novices”. Generally, clinicians saw CysC as the “right tool for the job” in a select set of clinical scenarios. These included clarifying true GFR when SCr accuracy was questioned, to assess kidney insults and AKI, and to guide renal drug-dosing.

Surprisingly, despite the known limitations of SCr, the majority of study participants remained somewhat ambivalent about the necessity of CysC testing. While CysC was perceived by all participants to estimate GFR more accurately than SCr, it did not appear clinicians desired de-implementation of SCr as standard of care [33]. Instead, CysC was often described as a helpful adjunct when SCr had known confounders or was thought to be otherwise inaccurate. Other more mainstream tests to corroborate kidney function (e.g., measured urine creatinine clearance) were perceived as excessively cumbersome, and the simplicity of a blood draw with CysC was viewed favorably. One mostly “invisible” role of CysC in the inpatient setting was as a tool that offered a definitive result to expedite workflow in various ways.

### Limitations

Several limitations of this study should be noted. First, this was a single-center study so findings are not necessarily generalizable. Currently few hospitals have rapid turnaround CysC testing [24]. Thus, ours was an exploratory effort to establish a baseline for future comparisons. Next, the study relies on self-reported data from clinicians and is subject to limitations including selective memory, fallibility in recall, and a general tendency to conflate actual behavior and ideal behavior. To ensure we reflected the broader context, we drew on results from our quantitative data on hospital CysC ordering patterns from 2011–2018 and the experience of team members with intimate knowledge of our study setting [24]. We did find a high degree of congruence across participant accounts of CysC ordering, although this is unlikely to represent completely shared opinion or knowledge of the topic.

While individually interviewing participants limited the number of total interviews feasible, the composition of our participant sample was informed by our previously published quantitative paper [24]. Our prior study found 47% of CysC tests were ordered for ICU patients, which necessitated adequate inclusion of intensivists [24]. Likewise, we previously found that the majority of patients with a CysC test ordered did not have a nephrology consult, so only one nephrologist was interviewed [24]. Notably, three senior nephrologists served on the investigative team (Rule, Kashani, Lieske) to ensure nephrologist input on interpretation of the findings. Finally, multiple pharmacists were interviewed since CysC is used in pharmacist-led dosing protocols for vancomycin at our institution [29, 30]. This study focused on clinical utilization of CysC, thus the perspective of laboratory medicine personnel were not reflected in these data. Such individuals would be able to comment on the logistical challenges with operationalizing rapid-turnaround CysC testing and reporting. It is essential to involve these individuals upfront during decisions about whether to introduce a new diagnostic test, its place in decision-making, and the feasibility of use. It was also beyond the scope of the current study to describe the place in practice of additional novel biomarkers outside of CysC, such as the functional biomarker β-trace protein or damage biomarkers such as neutrophil gelatinase-associated lipocalin (NGAL), tissue inhibitor metalloproteinase-2 (TIMP-2), and insulin-like growth factor binding protein 7 (IGFBP7). These are not available for routine clinical care at the study site thus clinicians would not be able to comment on their use in practice. Finally, the PI (Barreto) is a knowledgeable pharmacist and CysC researcher. Her participation and interest in this topic, enabled this in-depth study, but also may have introduced some degree of bias. Methodologic features designed to minimize the effect of this potential bias included the engagement of other study investigators including one co-investigator with a social science background rather than a medical background, interviews being conducted by other study team members in cases of pre-existing relationships between the PI and the participants, and routine dialogue with the multidisciplinary investigative team regarding individual assumptions and biases.

### Future directions

CysC is a useful adjunct to SCr for kidney function in the inpatient and outpatient setting [31]. After decades of a SCr-only strategy, clinicians now have a growing array of tools to monitor kidney function and injury at their disposal. While other examples exist, such as β-trace protein, β2 microglobulin, NGAL, TIMP-2, and IGFBP7, CysC may provide the most promise for rapid clinical utility [34–36]. Some have advocated that CysC is ready for a more widespread role in clinical use and at more hospitals [37]. While our aim was not to promote or refute the appropriateness of CysC testing, these data do highlight that with test availability there is a need for practitioner education. Our findings indicate that before CysC or any other such tests are implemented in clinical care, explicit education and practice guidance are needed to avoid confusion, waste, or harm. We suggest an educational program on CysC should address the following points:

1. CysC nonrenal determinants and independence from muscle mass
2. CysC and SCr units of measurements and equations
3. CysC kinetics in AKI relative to SCr
4. Use of CysC and SCr together for drug dosing of renally-eliminated medications; need for individualized drug dosing models rather than broad-application of new eGFR equations to historical dosing thresholds

Ideally the education would take a multimodal approach including informational learning modules with patient case examples, Grand-Rounds style lectures, and audit and feedback by experts on appropriate utilization and interpretation. Part of the education should be tailored to discipline and level of familiarity (novice, proficient or expert) with CysC. A well-rounded approach could facilitate uptake and appropriate use in clinicians who do not use the test because they are unaware of the value, or in those who lack confidence in the interpretation and application.

Direct information on the role of any of these tests alongside SCr and how to interpret their findings, particularly when they are ‘discordant,’ is critical to ensuring their safe and effective use. An electronic trigger system designed to consult nephrology in the presence of highly discrepant CysC and SCr values (i.e. if CysC is 2mg/L compared to SCr of 1mg/dL) could ensure “novice” or “proficient” clinicians receive support from “experts” when appropriate.

An important next step based on these data is to define the place for CysC testing among hospitalized patients and consider the protocolization of the test in specific scenarios. Based on our findings, we suggest the following situations as suitable for CysC use: (1) in patients with concerns regarding low muscle mass leading to decreased SCr production and over-estimation of kidney function, (2) AKI or renal recovery with an unknown trajectory, and (3) dosing of nephrotoxic or narrow therapeutic index, renally-eliminated medications. The key barriers and facilitators to CysC utilization identified in this study could provide a roadmap for implementing or expanding the use of CysC testing in the hospital. Most importantly, these would include the systems-based facilitators of rapid turnaround of results reporting (ideally <3hr), automated eGFR calculation, and organized education on the test. Nephrologists and pharmacists could serve as practice champions and a resource for other users.

## Conclusions

The findings of this study elucidate clinician approaches to CysC utilization and understanding of the test. While heterogeneity exists among clinicians in their views about the utility of CysC in inpatient care, there is a clear trend toward increased CysC usage to address the shortcomings of serum creatinine. Rapid results reporting is essential to ensure the test results retain clinical utility. These findings may assist other centers when determining whether and how to implement CysC testing. Future research should seek to refine clinical guidance for its utilization.

## Supporting information

S1 Appendix Semi-structured interview guide. (DOCX)
