# Supplementary material for: Clinician perspectives on inpatient cystatin C utilization: A qualitative case study at Mayo Clinic
Source: PLoS One. 2020 Dec 11;15(12):e0243618. doi: 10.1371/journal.pone.0243618 (PMC7732069; doi:10.1371/journal.pone.0243618)
Supplement: S1 Appendix — (DOCX) [file pone.0243618.s001.docx]

**S1 Appendix**. Semi-structured interview guide.

*Introduction:* Over the last ten years or so, we’ve seen some changes to how people approach kidney function assessment in hospitalized patients. One newer test, cystatin C, seems to be gaining traction in the hospital setting. We’ve looked at the Mayo data and seen an increase in use over the last decade or so in the hospital, and we want to talk to people from a variety of backgrounds who may be exposed to the test to understand more about it. We are interested in talking with you - as a clinician - to develop a better understanding of your impressions and your “first person” perspective.

*Housekeeping issue*: Before we begin, just want to note that we’d like to record this conversation. We plan to transcribe the tape and review the transcript as a team. The transcript will not include your name but we would like to capture some basic information about you to understand the background and experience that underlies your thoughts. Is that okay with you?

**

1. ***Context.*** To help me have a little the context for your comments, could you tell me briefly about the work you do, the patient population you deal with and how long you have been in practice?

*Probe:* If unclear, explicitly ask years of experience total, elsewhere and at Mayo Clinic, job role (e.g. physician, APP, pharmacist), environment – ICU, floor. *Emphasize that the focus remains on hospitalized patients so if they have a mixed practice, hopefully they can reflect on the inpatient aspect explicitly*

1. ***Standard of care:*** As I understand it, the current approach to assessing kidney function in hospitalized patients uses creatinine, and perhaps other tools. How do you feel creatinine meets your needs for that purpose?

*Probe: Examples of situations where creatinine works well, examples where it doesn’t work so well in the hospital*

*Probe: In situations where you feel like creatinine has some limitations, how do you*

*approach kidney assessment (e.g. offer specifics like reduced muscle mass, elderly,*

*amputation)? Have you used cystatin C or other tests or tools in those situations?*

**(Q3-4 for participants who do not use cystatin C, otherwise skip to Q5)**

1. ***Ideal state*:** In these cases where you’re not fully certain about the performance of creatinine to estimate GFR, what would be some aspects of an ideal test that would be important to you?
2. ***Non-use practices*:** We are seeing that some other clinicians are using cystatin C to assess kidney function for hospitalized patients at Mayo Clinic, perhaps to meet this need, what’s your take on that?

*Probe:* Why do you think that it’s happening? When do you think it started or what

catalyzed it?

*Probe:* What thoughts do you have about other people using cystatin C to assess kidney

function? *(specific inquiry about concerns)*

*Probe:* When someone checks it, how does having it in the medical record influence

decisions if at all? If you think about younger people or those at the front end of their career getting a test and just running with it, do you have any thoughts/concerns? Are there situations you’d be worried about with people using the test inappropriately?

**(Q5 and on for participants who have used cystatin C)**

1. ***History.*** Can you recall when you first became aware of Cystatin C?

*Probe:* How did it first come on your radar? (i.e. from a colleague, during residency/fellowship, CME, etc.) Are there certain groups of people or clinicians who brought it to your understanding?

1. ***Current practice.*** What is your understanding of the use of cystatin C in the hospital setting?

*Probe:* How has your understanding or comfort with the use of cystatin C evolved over

time?

1. ***Circumstances*.** In what circumstances do/would you use Serum cystatin C in the hospital?

*Probe:* If mention drug dosing – What drugs? How do you use cystatin C with the drug dosing guidelines that we provide? I imagine you’re pretty confident in how you’re thinking about it, but say for example for a new clinician trying to figure out when to use cystatin C, how would you advise them about the who and how?

1. ***Logistics.*** Are there any specific “barriers” to the use of cystatin C for your patients?

*Probes:* Institutional policies/procedures, electronic tools, turnaround time, EPIC eGFR reporting/calculations, education

*Probes:* Is there anything about the test availability that leads you to tend to recommend it?

1. ***Interpretation of results.*** Tell me about how you interpret the cystatin C-estimated GFR compared to the creatinine-estimated GFR?

Probe: If you have both the cystatin C eGFR and the creatinine eGFR available: If they are similar how do you approach decision-making, do you ever recheck it or? If they are different how do you think about/reconcile the results as a basis for patient care?

*Probe:* Can you walk us through a scenario, let’s say you had a patient who had a creatinine checked and the calculated eGFR was 80mL/min, but for unclear reasons the cystatin C was also checked and the reported eGFR was 30mL/min, how would you approach that clinically? Do you ever use the average of the two, some middle-ground?

1. ***Summary.*** Overall, what are the primary benefits you see for cystatin C testing in the hospital?

Probe: What are some of the concerns you have about using cystatin C testing in the hospital?

Probe: I know Mayo in Rochester has this, but there are a number of centers who don’t, do you have any thoughts on why they might not have it? Would you advocate for them getting it? If so, what would be your recommendations to them as to how to begin understanding it and using it in the hospital setting? I imagine there was a learning curve for you and probably similarly there would be for others.

1. ***What else?*** Is there anything we didn’t touch on that you think we should consider as we look at this issue?

(Thank them, plan to share the results and feed-back the findings.)
